# Use of Endo-Ovarian Tissue Biopsy and Pelvic Aspirated Fluid for the Diagnosis of Female Genital Tuberculosis by Conventional versus Molecular Methods

**DOI:** 10.1371/journal.pone.0098005

**Published:** 2014-05-21

**Authors:** Venkanna Bhanothu, Jane P. Theophilus, Roya Rozati

**Affiliations:** 1 Dept. of Zoology, Osmania University, Hyderabad, Andhra Pradesh, India; 2 Dept. of Reproductive Medicine, MHRT Hospital & Research Centre, Hyderabad, Andhra Pradesh, India; Fundació Institut d'Investigació en Ciències de la Salut Germans Trias i Pujol. Universitat Autònoma de Barcelona. CIBERES, Spain

## Abstract

**Background:**

Til date, none of the diagnostic techniques available for the detection of female genital tuberculosis (FGTB) are 100% accurate. We therefore, proposed to use the endometrial tissue biopsies (ETBs), ovarian tissue biopsies (OTBs) and pelvic aspirated fluids (PAFs) for the diagnosis of FGTB among infertile women by conventional versus molecular methods.

**Methodology/Principal Findings:**

A total of 302 specimens were collected both from 202 infertile women highly suspected of having FGTB on laparoscopy examination and 100 control women of reproductive age. Out of 302 specimens, 150 (49.67%) were ETBs, 95 (31.46%) were OTBs and 57 (18.87%) were PAFs. All specimens were tested by conventional techniques, later compared with multi-gene PCR for the detection of *Mycobacterium tuberculosis* (MTB) and correlated with laparoscopic findings. The presence of MTB DNA was observed in 49.5% of ETBs, 33.17% of OTBs and 5.44% of PAF specimens collected from highly suspected FGTB patients. All women of control group were confirmed as negative for tuberculosis. The conventional methods showed 99% to 100% specificity with a low sensitivity, ranging from 21.78% to 42.08% while hematoxylin and eosin staining showed a sensitivity of 51.48%. Multi-gene PCR was found to have much higher sensitivity of 70.29% with MTB64 gene, 86.63% with 19 kDa antigen gene at species and TRC4 element at regional MTB complex and 88.12% with 32 kDa protein gene at genus level. The specificity of multi-gene PCR was 100%. Compared with culturing and Ziehl-Neelsen's staining, multi-gene PCR demonstrated improvement in the detection of FGTB (χ^2^ = 214.612, 1 df, McNemar's test value <0.0001).

**Conclusions Significance:**

We suggest site specific sampling, irrespective of sample type and amplification of the 19 kDa antigen gene in combination with TRC4 element as a successful multi-gene PCR for the diagnosis of FGTB and differentiation of mycobacterial infection among endo-ovarian tissue biopsies and PAFs taken from infertile women.

## Introduction

Female Genital Tuberculosis (FGTB) which is commonly implicated as a cause of infertility [1]–[5], is symptom-less, rare disease with non-specific, mild clinical pictures and low index of clinical suspicion. There are no reliable confirmatory investigative procedures to ascertain the cause of infertility [4], [5]. Genital tuberculosis represents 15–20% of extra pulmonary tuberculosis [6] and remains to be a major health problem in developing countries [7]. The incidence of infertility in genital TB worldwide varies from 10–85% [5]–[9]; it is endemic in India, with an incidence of 58% [10] and majority are in same reproductive age group (15–45 years) [11]. In 80–90% cases, it affects women with menstrual irregularities accounting for about 27% of manifestations of FGTB [12], this rate is observed to be higher among patients with tubal factor infertility (39–41%) [13].

### Diagnosis and limitations

*M. tuberculosis* (MTB) is a facultative intracellular acid-fast gram-positive pathogenic bacterium capable of producing both a progressive disease and an asymptomatic latent infection [14], [15]. It generates worrying effects, causing irreversible damage to the fallopian tube with potential consequences of causing infertility and making it untreatable both by medical and surgical methods [16], [17]. It is estimated that at least 11% of the patients lack symptoms and FGTB is often detected in diagnostic workup of women attending infertility clinics [18]. Thangappah *et al* revealed that 57% of infertile women in whom the presence of TB was suspected on clinical grounds had a positive endo-TB-PCR test, whereas only 9.5% had a positive test with no clinical ground for suspicion [19]. Though, the actual incidence may be under-reported due to asymptomatic, varied clinical presentations, diverse imaging, transforming laparoscopic results and a mixed bag of bacteriological and serological tests [4], [5]. Therefore, a high degree of suspicion assisted by intensive investigation is significant in the diagnosis of the disease. A positive chest X-ray for healed or active pulmonary tuberculosis, contact history, elevated erythrocyte sedimentation rate (ESR), positive tuberculin skin test and sampling by laparoscopy may specify the need for further investigations [20]–[22]. Studies on serial sections of tissues are needed because the lesions are frequently erratic and even in that case also positive endometrial culture for TB was found only in 25% of cases of tuberculous endometritis as the endometrium is often focal and moreover, due to the cyclical shedding of the endometrium, granulomas do not have enough time to form [23], [24]. In such cases, the granulomatous endometrium may not show evidence of tuberculosis in all the cycles. Further the conditions like, oophoritis (inflammation of the ovaries) are also often seen in combination with salpingitis (inflammation of the fallopian tubes). Usually, it is a rare condition, following haematogenous spread and causing infertility. Since, there is no way to take the fallopian tubes out, sampling from the ovaries and endometrium was suggested for the detection of FGTB [25]. Use of menstrual blood for bacteriologic or molecular diagnosis has been recommended [26] but was reported to show low sensitivity [27].

Conventional/phenotypic methods have slow and low detection rates and have limitations due to secondary nature of genital tuberculosis. Sampled sites may not represent the infected area and the infected site can be simply missed due to sparse number of paucibacillary nature of mycobacteria [19]. According to tissue reactions, in those having tuberculosis may at times be atypical and bacteriologically mute [18]. Thus, these methods have poor sensitivity and specificity for the diagnosis of FGTB throughout the sub-clinical stages. On the other side, a range of PCR techniques have been mechanized for the detection of specific nucleic acid sequences of MTB and other mycobacteria [28]. Identification of genes encoding the virulence determinants, available targets in genome and highly expressing factors are currently important biomarkers for the detection of FGTB. Biological, molecular and immunological studies have resulted in identification of more than 33 different useful proteins, some of which are specific to MTB or MTB complex [29]. Out of which, four genes has been selected for our study considering their importance in the analysis of ethiopathogenesis and diagnosis of FGTB. In the present study, we report the efficacy of an amplification format based on the identification of the causative agents among highly suspected cases of FGTB in India at the genus (32 kDa protein/MPT59 α (alpha)–antigen gene), MTB complex (MPB64 gene), regional specific MTB complex (TRC4 element) and species levels (19 kDa antigen) in determining the tubercular aetiology of female infertility. Further, we have attempted to evaluate the diagnostic competence of multi-gene/multi-primer PCR (multi-gene PCR) vis-à-vis laparoscopic findings, besides establishing the appropriateness of using multiple samples, namely endometrial tissue biopsies (ETBs), ovarian tissue biopsies (OTBs) and pelvic aspirated fluids (PAFs) for accurate diagnosis of FGTB.

## Materials and Methods

### Ethical Statement

The study protocol was in compliance with the Declaration of Helsinki, approved by the ethics committee of MHRT Hospital and Research Centre, Hyderabad, India. Informed consents from the patients were taken by our institution through the ethical committee, since patient's samples were obtained by operative laparoscopy.

### Study design

A prospective case-control study was set in the Zoology Modular Lab, CFRD, Osmania University, Hyderabad, India. During the period of our study (September 2006–February 2014), the samples from infertile women visiting the gynaecology clinics at two collaborating centres in Hyderabad were analysed. All patients met the definitive/confirmed **inclusion criterion** for selecting FGTB suspected cases indicating clinical symptoms such as infertility among reproductive age group (18–40 years), pelvic pain, scanty menstruation with irregular periods, dysmenorrhoea, oligomenorrhea, amenorrhoea, general malaise and menorrhagia leading to the abortions. Radiological findings may or may not be indicating the tuberculosis. Laparoscopic findings [30] including; 1) evocative diagnosis of TB (occurrence of caseation, granuloma/tubercles and/or beaded/thickened tubes; 2) possible diagnosis of TB (hydrosalpinx, peritubal/periovarian adhesions, tubo-ovarian mass without frank tubercles/caseation; 3) incidental findings (pelvic pathology other than pelvic inflammatory disease including fibroid uterus, endometriosis, polycystic ovaries); 4) normal findings. Histopathological findings [31], [32] indicating chronic inflammation or lesions such as proliferative solid epitheliod granulomas, dense polymorphornuclear cells, lymphocytic infiltrations, giant or beaded cells, enlarged lymphoid cells and accumulation of plasma and spindle cells. Demonstration of tubercle bacilli in culture, Z–N staining of menstrual blood fluids, pelvic aspirated fluids, endometrial curetting, endometrial tissue biopsies and ovarian tissue biopsies were considered. However, the most important positive response of FGTB suspected cases to the anti TB therapy is failure to support fertility and improvement in the response can be seen after anti-tubercular chemotherapy [33], by means of the methods like ovulation induction (OI), intra uterine injection (IUI), intracytoplasmic sperm injection (ICSI), in-vitro fertilization (IVF) in 3–4^th^ cycles and appropriate surgical interventions. **Exclusion criteria** were the following: Women above 40 years of age, symptoms suggesting pulmonary tuberculosis (TB)/extra pulmonary TB except infertility, with normal abdominal and vaginal examinations, pregnant and nursing women, patients with severe psychiatric dysfunctions, endocrine problems, sexual disorders, multiple sclerosis or other autoimmune disorders, pulmonary infections, HIV co-infection, women with diabetes, malnutrition and other medical disorders like hypertension, peritoneal adhesions due to previous abdominal surgery, infertility due to male factors and abnormality in ovulations.

Information on the general, obstetric and gynaecological details including family history, marital status, age at menarche, length of menstrual cycle, associated symptoms, duration and amount of blood loss, duration of infertility, and socio-demographic details like social status, occupation, lifestyle, age, body mass index (BMI), limited information on dietary and nutrition factors were obtained. Apart from routine examinations, laparoscopy and hysteroscopy were performed at infertility workup as and when needed. All subjects were HIV negative and negative for pulmonary TB on the basis of complete history, physical examinations; chest X-ray, lung plain X-ray and by appropriate tests such as tuberculin skin test [20]. Details of hystero-laparoscopy findings like unilateral or bilateral tubal block with hydrosalphinx, omental adhesions, frozen pelvics, tubo-ovarian masses, tubercular salphingitis, beaded tubes and tubercles were noted. Beaded appearance of tubes, frank tubercules on uterus and pelvic mass in variable combination aroused a suspicion. Constitutional symptoms such as sweating, increase in temperature and weight loss were not major complaints while local organ dysfunction manifested in amenorrhea, omental adhesions and bilateral tubal blockage are seen on hysterosalpingographic study. The diagnosis was made based on morphological [20] and molecular investigations.

All samples collected during laparoscopy were examined by hematoxylin and eosin (H & E) staining [34]–[36], Ziehl-Neelsen's (Z–N) staining for acid fast bacilli (AFB) [36]–[38], cultured on Löwenstein–Jensen (L-J) egg media [38]–[40] and MTB specific multi-gene PCR method [18], [19], [41]–[50] by which FGTB was confirmed and correlated with laparoscopic findings. All specimens tested by conventional/phenotypic methods were later compared with multi-gene PCR method using four sets of primers for the detection of MTB in a single tube- single step reaction. Primer selections for the multi gene PCR study were done based on local characteristics of the strains and their importance in diagnosis of the disease. The nucleotide sequences of primers used for the detection of *Mycobacterium* in this study were described and validated as diagnostic markers in the past (described in **Table 1**) [18], [19], [41]–[50]. *M. tuberculosis* (ATCC 35836) reference stain isolates provided by Dept of Microbiology, Nizam's Institute of Medical Sciences, Hyderabad (India) were used as controls in each assay.

**Table 1:** Details of genes of *Mycobacterium tuberculosis* Complex.

S.No	Gene	Forward primer	Reverse primer	Length & Size (bp)	Reported annealing temp.	Function	Conclusion	Ref.
1	19 kDa Antigen gene	5′TCTTTCCGGATGTTCAAGCA 3′	5′TGACGTTCTGGTCCTTACC3′	20; 131	58, 68	It acts as antigen for cellular and humoral arms of the adaptive response. Involved in suppression of growth and apoptosis of infected cells.	Secreted nature can contribute to its serological immuno-dominance by enhancing its accessibility in a native form for B-cell recognition.	41, 42
2	TRC4 Element	5′GACAACGACGTGCGCCTACT 3′	5′GACCGAATTAGCGTAGCTCC 3′	20; 173	57, 58	It is from a very essential region of *M. tuberculosis* genome participating in recombination	Ideal target for PCR assays to identify *M. tuberculosis*; especially in strains carrying no copies of IS6110 in extra pulmonary patients	19, 43, 44
3	MPB64 Antigen gene	5′TCCGCTGCCAGTCGTCTTCC 3′	5′GTCCTCGCGAGTCTAGGCCA 3′	20; 240	55,60	Highly immunogenic antigen and found in active cultures	This polymeric epitopes can be a good biomarkers for serodiagnosis	18, 45, 46
4	32 kDa Protein gene	5′TTCCTGACCAGCGAGCTGCCG 3′	5′CCCCAGTACTCCCAGCTGTGC 3′	21;506	68, 71	Abundantly secreted, catalyses in forming mycobacterial cell wall assembly	This antigen would provide a target, which is universally present.	47–50

T: thymine; A: adenine; G: guanine; C: cytosine.

### Processing of Tissue Biopsy

The specimens (like ETBs, OTBs and PAFs) collected from the lesions over the endometrium, ovaries and pelvis were mixed with sterile normal saline, transported in sterile vials to the laboratory and processed as per standard protocols [18], [19], [51]–[54]. Whole specimens were washed with saline and centrifugation at 6000 rpm for 10 min at 4°C. Supernatant were discarded and the pellets were reconstituted with 1 ml of sterile Tris Buffered Saline (1X TBS; pH-7.4). Each specimen (from TBS) was divided into two portions-one kept for decontamination and concentration for subsequent studies such as Z-N staining, culturing on L-J media and extraction of DNA for multi-gene PCR method, and the other portion kept was used for histopathology, RNA and protein extraction.

### Decontamination and Concentration (D & C)

All specimens were decontaminated and concentrated by modified HS–SH procedure [55]. About 200 µl of tissue biopsies or scrapings were minced and grinded well using tissue homogenizer and mixed with 200 µl of 7% (w/v) NaCl and 200 µl of 4% (w/v) NaOH in a sterile eppendorf centrifuge tube. Then, the tubes were incubated at 37°C for 30 min. The content was neutralized with sterile normal saline; to make the total volume to 2 ml was mixed for 5 seconds and centrifuged at 12,000 rpm for 15 min at 4°C using aerosol proof shields. The supernatant was discarded into a splash-proof container with a tuberculocidal solution and the pellet was resuspended in 200 µl of sterile TBS buffer and mixed for 5 seconds. Homogenised tissue sediments were used for culturing on L-J medium, Z-N staining and for the isolation of mycobacterial DNA.

### Histopathological Examination

**Figure 1:**
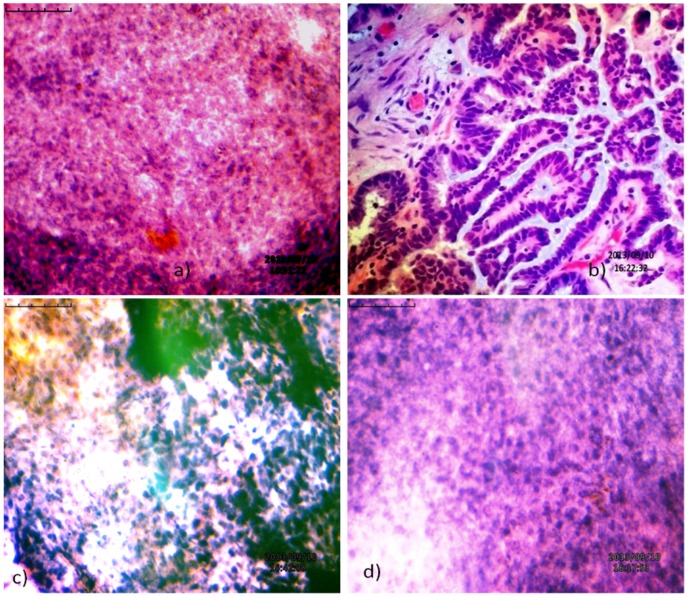
Histopathological examination of endo-ovarian tissue biopsy. a) Endometrial tissue biopsies from the surface resemble those of cervical microglandular hyperplasia with beaded spindle-cells; b) Beaded and malignant lymphoid cells were observed in ovarian tissue biopsies; c) Appearance of spindle-cells with superficial strips of positive endometrial glands and stroma; d) Lymphocytic infiltrations and initial stages of granulomatous were observed. **Note**: The microscopic studies were carried out with tissue biopsies and aspirated fluids containing tissue pieces. Thereafter, the slides were viewed under bright field (40X), Inverted microscope. The contrasts of the photographs are changed to improve the visibility.

Thin slices of the processed tissue biopsies and PAFs were placed onto the slides and kept for air drying at room temperature. Tissue specimens were fixed with buffered formalin (10%), cleaned with xylene and dehydrated with absolute alcohol (100%). The slides were again fixed with ethyl alcohol (95%), washed with water and dried. Then, the slides were stained with Weigert's iron hematoxylin, washed with water, differentiated with acid alcohol (1%) and alcohol (95%) and washed with water. Following, the slides were counterstained with eosin [34]–[36], dehydrated with increasing gradients of alcohol, cleaned with xylene and mounted. Mounted slides were viewed under bright field (40x), Inverted Biological Microscope (BLM-290, BestScope, China). The presence of caseating granulomas surrounded by epitheloid cells, malignant lymphocytic infiltrations, beaded plasma cells and giant polymorphornuclear cells were diagnostic of FGTB [31], [32] (shown in **Figure 1**).

### Ziehl-Neelsen's (Z-N) staining of tissue sediments for AFB

**Figure 2:**
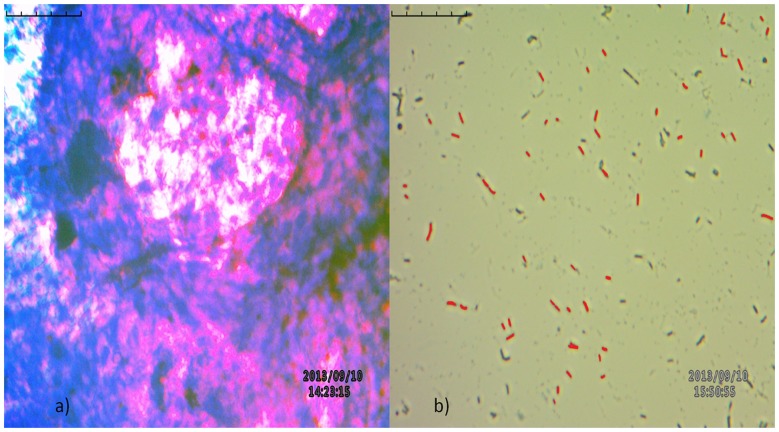
Z-N staining of endo-ovarian tissue biopsy and cultures in the detection of acid fast bacilli (AFB). a) Red/pink colour rod like beaded structures were observed in the tissue biopsy; b) Red/pink colour rod like structures were observed on pale blue background in the cultures.

About 50 µl of the decontaminated and concentrated endo-ovarian tissue sediments were spread onto the slides, kept air dried for 10 minutes at 60°C and heat-fixed for 10 minutes at 90°C. The slides were then stained with carbolfuchsin, decolorized by acid alcohol and counterstained by methylene blue and rinsed with water to remove excess methylene blue. Stained slides were viewed under Inverted Biological Microscope. The portion of smear that stained pink/red on pale blue background was noted as *Mycobacterium* [36]–[38] (shown in **Figure 2**).

### Culture of tissue sediments

About 50 µl inoculum of the decontaminated and concentrated endo-ovarian tissue biopsy sediments were taken and applied rapidly on L-J egg medium slants using sterile micropipette. Slant and bottled culture media were incubated in a horizontal plane until the inoculum was absorbed. The culture tubes were then incubated at 37°C for 6–8 weeks until heavy growth was obtained. Cultures were stained by Z-N staining for confirmation of AFB growth [38], [39]. Standard Precautions [39], [40] and Institutional guidelines have been followed in handling all items contaminated with blood and other body fluids.

### Quality control

Reagents were aliquoted and each aliquote was used only once. Sterile microcentrifuge tubes and PCR tubes were used for the PCR assay. Reagent preparation, DNA extraction, DNA amplification and detection were performed in separate rooms to avoid cross-contamination of amplicons. A positive control was included in each test and distilled water was included as a negative test control. All these investigations have been conducted meticulously with appropriate controls, replications, large sample size and with different combination of experiments. The recommendations and regulations of the Clinical and Laboratory Standards Institute (CLSI, 2001 [40]) were followed for quality control and standards.

### DNA Extraction and Purification

All the decontaminated and concentrated endo-ovarian tissue sediments were centrifuged at 8,000 g for 10 minutes and 180 µl of 2X TE buffer (20 mM Tris-HCl, 2 mM EDTA, pH 8.0) containing 1% sodium dodecyl sulfate (40 µl from 10% SDS), 2.4% Triton X-100 was added to the pellet for lysis of gram-positive bacteria [56], 20 mg/ml of lysozyme was subsequently added, mixed and incubated for 30–60 min at 37°C. 25 µl of Proteinase K (20 mg/ml) was added and mixed by vortexing and is incubated in dry bath at 56°C for 1 to 3 hours until complete lysis was achieved (provided by DNASure Tissue mini kit from Genetix Biotech Asia Pvt. Ltd, New Delhi, India). Then, appropriate condition for binding of DNA to the silica membrane in the DNASure Tissue Mini Kit Columns was achieved by the addition of chaotropic salts and ethanol to the lysate. The binding process is reversible and specific to nucleic acids. Contaminations were removed by subsequent washing with two different buffers according to manufacturer instructions. Pure genomic DNA was finally eluted under low ionic strength conditions in a slightly alkaline elution buffer. The purity of DNA was checked on 0.8% agarose gel electrophoresis, incorporated with ethidium bromide. The bands in the gel were photographed under a Bio-Rad Gel documentation system and quantified.

### Multi-gene/Multi-primer PCR method

**Figure 3:**
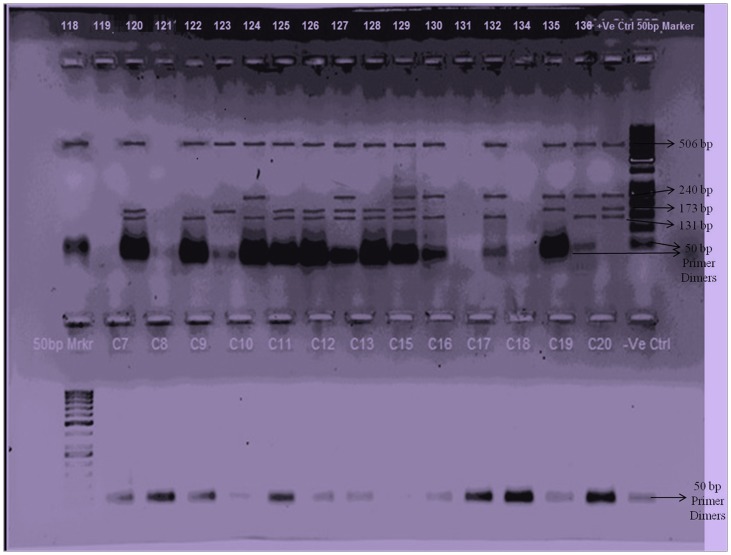
2.5% Agarose Gel Electrophoresis was carried out with Multi-gene/multi-primer PCR products. Lanes in the first row (118 to 136) were loaded with PCR products of FGTB patients; Lanes in the second row (C7 to C20) were loaded with PCR products of control patients; Lane +Ve Ctrl was loaded with positive Reference Strain (*M. tuberculosis*, ATCC 35836); Lane –Ve Ctrl was loaded with negative control (H_2_O); Lane 50 bp marker was loaded with 50 base pair (bp) molecular weight ladder (50 bp size of product starts from the bottom side of gel and ends with 650 bp product on the top/upper side of the gel), The band corresponding to 131 bp was noted as 19 kDa antigen gene, 173 bp was noted as TRC4 element, 240 bp was noted as MPB64 gene, 506 bp was noted as 32 kDa protein/MPT59 α- antigen gene. Primer dimers were also noted at the bottom during the end of sample run.

Multi-gene PCR was performed with 20–30 ng extracted DNA, and DNA amplification with 5 U Taq DNA polymerase (Bangalore Genie, Bangalore, India), 10 mM deoxyribonucleoside triphosphates (Bangalore Genie, Bangalore, India) and 13.5 pmol each primer (Bioserve Biotechnologies (India) Pvt Ltd, Hyderabad, AP, India) in a final volume of 50 µL. Two looped touchdown multi-gene PCR program; each with 25 cycles was followed. In the first loop, the template DNA was initially denatured at 95°C for 5 minutes then denatured at 94°C for 45 seconds, annealed at 68°C for 45 seconds, extended at 72°C for 45 seconds and continued for total of 25 cycles. In 2^nd^ loop; DNA was denatured at 94°C for 45 seconds, annealed at 58°C for 45 seconds, extended at 72°C for 45 seconds and continued for total of 25 cycles with final extension at 72°C for 15 minutes. The PCR amplification was done using master cycler gradient PCR system (Eppendorf, Hamburg, Germany). PCR products were subjected to electrophoresis in a 2.5% agarose gel incorporated with ethidium bromide, along with Gene Rule 50 bp DNA ladder/molecular weight marker. The electrophoresis was carried out at a constant voltage (110 V) for one hour. The bands in the gel were photographed under a Gel Doc-XRT with image lab software (Molecular Image, Bio-Rad, Hercules, CA, United States of America). As, we find clear and accurate banding patterns by agarose gel electrophoresis, sequencing of the PCR product was not suggested. The results of case-control groups were compared with reference strain (shown in **Figure 3**).

### Statistical analysis

The sensitivity, specificity, positive predictive values and negatives predictive values were calculated using standard formulae [45] for diagnostic accuracy. The association between various quantitative variables (age, age at menarche age, body mass index and duration of infertility among infertile women highly suspected with FGTB and control women) was analysed by using SPSS v 20 for Windows (IBM SPSS Statistics 20, Chicago, USA). The data was presented as mean ± standard deviation (SD). Association among qualitative variables (findings of conventional and molecular methods among infertile women highly suspected with FGTB and control women) were statistically analysed by Pearson Chi-square test or Fisher's exact test or McNemar's test, as needed. The significant differences in the positive rates of different methods were analysed. Data were considered statistically significant if p value was less than 0.05.

## Results

The present study assesses the role of multi-gene PCR in the diagnosis of FGTB using ETBs, OTBs and PAF samples, and correlated with laparoscopic findings. A total of 302 specimens were taken for study which included samples from 202 infertile women highly suspected of having genital tuberculosis on laparoscopic examination and samples from 100 control women (without TB) of reproductive age.

The mean age of the subjects was 28.54±4.46 years, mean duration of infertility was 3.92±3.03 years and BMI was 24.36±1.47. The majority of patients (77.23%) presented primary infertility, 46 (22.77%) with secondary infertility and 69 (34.15%) women experienced abortion. Apart from infertility, 125 (61.88%) patients had other menstrual complaints such as dysmenorrhoea in 94 (46.53%) women and abdominal/pelvic pain in 31 (15.34%) women. 63 (31.19%) patients had menstrual irregularities such as oligomenorrhea (12.87%), amenorrhea (8.91%), general malaise (4.95%) and menorrhagia (4.45%) among infertile women were given in **Table 2**. All FGTB patients were negative for chest X-ray and 42 (20.79%) patients were positive for Mantoux test. Erythrocyte sedimentation rate (ESR) was elevated in all FGTB patients. All the modalities of treatments such as ovarian induction, intra uterine injection, intra cytoplasmic sperm injection, in-vitro fertilization in 3^rd^–4^th^ cycles and appropriate surgical intrusions were unsuccessful to support fertility prior to anti-tubercular chemotherapy. Favourable infertility outcomes following the anti-tubercular chemotherapy was reported among FGTB suspected cases [57]. Further, efforts were made to examine the efficacy of diagnostic tests only rather than the treatment. Laparoscopy usually detects macroscopic changes such as beaded tubes in 139 (68.81%) women, tubal block with hydrosalphinx in 119 (58.91%), tubercular salpingitis in 97 (48.01%), omental adhesions in 78 (38.61%) and multiple tubercules in 72 (35.64%) women were recorded in the case group [9]. A standard protocol of investigations revealed a number of causes for fertility deprivation.

**Table 2:** Demographic and clinical findings of FGTB cases and control groups (n = 302).

Characteristics	Infertile women suspected with FGTB (n = 202) cases	Control group (n = 100)
Age (Years)	28.54±4.46	27.59±4.62
Age at menarche (Years)	12.49±1.02	12.37±0.93
Body mass index (kg/m2)	24.36±1.47	24.05±1.68
Infertility symptoms		
Duration of infertility (Years)	3.92±3.03	0.174±0.184
Primary infertility [n (%)]	156 (77.23)	0 (0)
Secondary infertility [n (%)]	46 (22.77)	0 (0)
Proven fertile [n (%)]	0	100 (100)
Abortion [n (%)]	68 (33.66)	0 (0)
Menstrual irregularity		
Dysmenorrhoea [n (%)]	94 (46.53)	0 (0)
Mild [n (%)]	54 (26.73)	0 (0)
Moderate [n (%)]	29 (14.35)	0 (0)
Severe [n (%)]	17 (8.41)	0 (0)
No dyspareunia & dysmenorrhoea [n (%)]	52 (25.74)	90 (90)
Abdominal pain [n (%)]	31 (15.34)	0 (0)
Oligomenorrhea [n (%)]	26 (12.87)	3 (3)
Amenorrhea [n (%)]	18 (8.91)	0 (0)
General malaise [n (%)]	10 (4.95)	0 (0)
Menorrhagia [n (%)]	9 (4.45)	7(7)

**Note**: Some patients had more than one abnormal finding; Data are presented as mean ± Standard Deviation (SD); n: number of patients; %: percentage.

**Figure 4:**
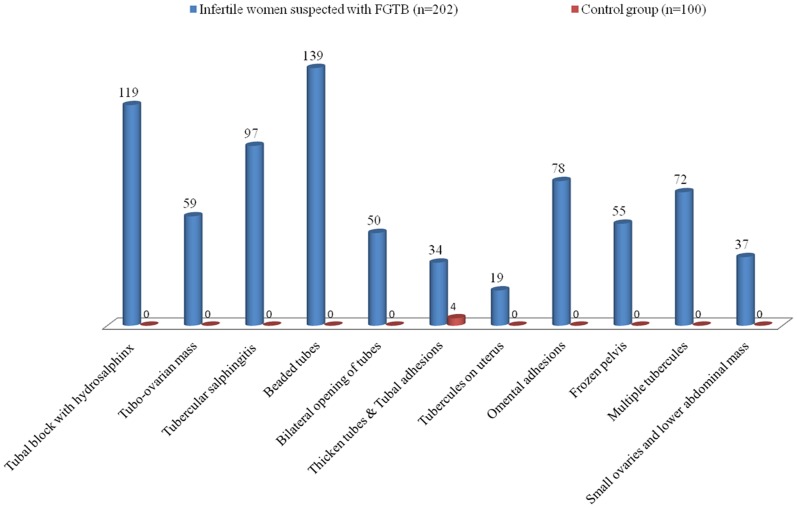
Laparoscopic/Hysteroscopic findings of infertile women suspected with female genital tuberculosis (FGTB) cases and control groups. Note : Some patients had more than one abnormal finding.

A total of 100 specimens were collected from control women (n = 100) of reproductive age (18–40 years). Women who attended the same clinic for other gyaecological disorders, tubal sterilization and laparoscopy for menorrhagia were selected as controls. All control women (negative for pulmonary tuberculosis) were fertile, negative for chest X-ray and laparoscopically confirmed to be without FGTB. Of which 27 (27%) were ETBs, 27 (27%) were OTBs and 46 (46%) were PAFs. All the women in control group (without TB) were asymptomatic with mean age of 27.59±4.62 years, mean duration of infertility was 0.174±0.184 years and BMI was 24.05±1.68. Menstrual irregularities such as oligomenorrhoea (3%) and mild menorrhagia (7%) were seen in the control women. 13 women in control group were positive to Mantoux test. Only thicken tubes and tubal adhesions were observed in 4 (4%) control women on laparoscopic examinations (**Figure 4**). None of the patients in our study reported family history of tuberculosis.

Out of the 202 specimens collected from highly suspected cases of FGTB, 123 (60.89%) were ETBs, 68 (33.66%) were OTBs and 11 (5.44%) were PAFs. Of the 123 (60.89%) ETBs, 57 (28.21%) were positive on H & E staining, 37 (18.31%) by culturing on L-J egg medium and 35 (17.32%) were AFB positive on Z-N staining of culture. Out of 123 ETBs, 97 (48.02%) were positive with 19 kDa antigen (131 bp) gene and 100 (49.50%) were positive by TRC4 (173 bp) repetitive element and MPT59 α -antigen (506 bp) gene (32 kDa protein) on multi-gene PCR. Of the 68 (33.66%) OTBs, 42 (20.79%) were positive with H & E staining, 41 (20.29%) by culturing on L-J egg medium and 39 (19.3%) were AFB positive with Z-N staining of culture. Subsequently, 67 (33.17%) were positive with 19 kDa antigen gene and 32 kDa protein gene by multi-gene PCR. It is also reported that, 5 (2.47%) out of 11 (5.44%) PAFs were positive with H & E staining and 7 (3.46%) samples were culture positive on L-J egg medium and also found AFB positive with Z-N staining. All PAFs samples (5.44%) were positive with 19 kDa antigen gene and 32 kDa protein gene by multi-gene PCR (**Table 3**). While, out of 202 specimens taken from suspected cases of FGTB, 175 (86.63%) specimens were positive for 19 kDa antigen gene (MTB species-specific antigen gene) and TRC4 repetitive element (South Indian MTB complex specific). 142 (70.3%) specimens were positive for MPB64 antigen (240 bp) gene (MTB complex specific). 178 (88.12%) endo-ovarian tissue biopsies and pelvic aspirated fluid specimens were positive with 32 kDa protein gene by multi-gene PCR method.

**Table 3:** Findings using conventional and molecular methods among infertile women and control group (n = 302).

Characteristics	Suspected cases of FGTB				Control Group without TB			
Type of samples	ETBs [n (%)]	OTBs [n (%)]	PAFs [n (%)]	Total [n (%)]	ETBs [n (%)]	OTBs [n (%)]	PAFs [n (%)]	Total [n (%)]
Methods/Distribution of Samples	123 (60.89)	68 (33.66)	11 (5.44)	202 (100)	27(27)	27(27)	46 (46)	100 (100)
H & E Staining	57 (28.21)	42 (20.79)	5 (2.47)	104 (51.48)	1(1)	0 (0)	0 (0)	1(1)
AFB+Ve on Z-N Staining of tissue sediments	12 (5.94)	29 (14.35)	3 (1.48)	44 (21.78)	0 (0)	0 (0)	0 (0)	0 (0)
L-J Egg medium	37 (18.31)	41 (20.29)	7 (3.46)	85 (42.08)	0 (0)	1(1)	0 (0)	1(1)
AFB+Ve on Z-N Staining of culture	35 (17.32)	39 (19.30)	7 (3.46)	81 (40.1)	0 (0)	0 (0)	0 (0)	0 (0)
19 kDa (131 bp)	97 (48.02)	67 (33.17)	11 (5.44)	175 (86.63)	0 (0)	0 (0)	0 (0)	0 (0)
TRC4 (173 bp)	100 (49.50)	66 (32.67)	9 (4.45)	175 (86.63)	0 (0)	0 (0)	0 (0)	0 (0)
MPB64 (240 bp)	71 (35.14)	63 (31.18)	8 (3.96)	142 (70.3)	0 (0)	0 (0)	0 (0)	0 (0)
32 kDa (506 bp)	100 (49.50)	67 (33.17)	11 (5.44)	178 (88.12)	0 (0)	0 (0)	0 (0)	0 (0)

**Abbreviations**: n: number of patients; %: percentage; FGTB: Female Genital Tuberculosis; ETBs: Endometrial tissue biopsies; OTBs: Ovarian tissue biopsies; PAFs: Pelvic aspirated fluids; H & E staining: Hematoxylin and Eosin; L-J egg medium: Lowenstein-Jensen; AFB+Ve: Acid fast bacilli positive; Z-N staining: Ziehl-Neelsen's; PCR: Polymerase Chain Reaction; 19 kDa (131 bp): 19 kDa antigen gene; TRC4 (173 bp): TRC4 element; MPB64 (240 bp): MPB64 antigen gene; 32 kDa (506 bp): 32 kDa protein (MTP-59 α-antigen) gene.

The multi-gene PCR detection rate for the disease was the highest of all the other methods as it detected, 178 (58.94%) out of the 302 endo-ovarian tissue specimens, 104 (34.43%) specimens collected from infertile women and 1 (0.33%) ETB from control woman was positive on H & E staining [18], [19], [47]. AFBs were positive in 44 (14.57%) D & C tissue specimens collected from infertile women and 1 (0.33%) OTB from control woman was positive by culture on L-J egg media. Out of 85 culture positive cases, 81 (26.82%) FGTB cases were found AFB positive by Z-N staining. All control women were found negative for tuberculosis with endometrium, ovaries and pelvis. The agreement between mycobacterial growth on L-J egg media and diagnosis of FGTB by multi gene PCR were examined using four sets of primers (**Table 4**). Of the 86 culture positive samples, 84 (97.68%) were positive by multi gene PCR using 19 kDa antigen gene and TRC4 element, 78 (90.7%) were positive by MPB64 antigen gene, whereas 85 (98.83%) were positive by 32 kDa protein gene. Out of 216 culture negative samples, 91 (42.13%) were positive and 125 (57.87%) were negative by multi-gene PCR using 19 kDa antigen gene and TRC4 element, 64 (29.63%) were positive and 152 (70.37%) were negative by using MPB64 antigen gene, and 93 (43.06%) were positive and 123 (56.94%) were negative by using 32 kDa protein gene.

**Table 4:** Agreement between culture (L-J egg media) and multi gene PCR results using four set of primers.

Multi gene PCR/Characteristics		Total [n = 302 (%)]	L-J Egg medium	
			Positive [n (%)]	Negative [n (%)]
			86 (28.47)	216 (71.5)
19 kDa (131 bp)	Positive	175 (57.95)	84 (97.68)	91 (42.13)
	Negative	127 (42.05)	2 (2.32)	125 (57.87)
TRC4 (173 bp)	Positive	175 (57.95)	84 (97.68)	91 (42.13)
	Negative	127 (42.05)	2 (2.32)	125 (57.87)
MPB64 (240 bp)	Positive	142 (47.02)	78 (90.7)	64 (29.63)
	Negative	160 (52.98)	8 (9.3)	152 (70.37)
32 kDa (506 bp)	Positive	178 (58.94)	85 (98.83)	93 (43.06)
	Negative	124(41.06)	1 (1.62)	123 (56.94)

**Abbreviations**: n: number of patients; %: percentage; H & E staining: Hematoxylin and Eosin; L-J egg medium: Lowenstein Jensen; AFB+Ve: Acid fast bacilli positive; Z-N staining: Ziehl-Neelsen's; PCR: Polymerase Chain Reaction; 19 kDa (131 bp):19 kDa antigen gene; TRC4 (173 bp): TRC4 element; MPB64 (240 bp): MPB64 antigen gene; 32 kDa (506 bp): 32 kDa protein (MTP-59 α-antigen) gene.

Disparity in the detection of FGTB by Z-N staining for AFB positive, histopathological evidence of tuberculosis infection and detection of mycobacteria using culture and multi-gene PCR among infertile women and control groups were described in **Table 5**. Fisher's exact test and McNemar's test were applied among FGTB cases and control group to ensure the TB infection using conventional (AFB, culture and histopathology) and molecular methods. The p-value was highly significant (Pearson χ^2^ = 214.612, 1 df, Fisher's exact test value <0.0001, McNemar's test value <0.0001) with multi gene PCR, whereas moderate agreement (Pearson χ^2^ = 55.418, 1 df, Fisher's exact test value <0.0001, McNemar's test value  = 0.338) was reported with culture and mild agreement (Pearson χ^2^ = 75.167, 1 df, Fisher's exact test value <0.0001, McNemar's test value  = 0.779) was observed with histopathology. Further, the conventional methods showed 99% to 100% specificity with a low sensitivity, ranging from 21.78% to 42.08% while H & E staining showed a sensitivity of 51.48%. However, multi-gene PCR method was found to have a much higher sensitivity of 70.29% for MTB64 gene, 86.63% for 19 kDa antigen gene and TRC4 element and 88.12% for 32 kDa protein gene [47], [58]. The specificity of multi-gene PCR was 100%. The conventional/phenotypic methods had 98.83% to 100% positive predictive value with a lower negative predictive value, ranging from 38.75% to 45.83% whereas H & E staining had a negative predictive value of 50.25%. Multi-gene PCR method was found to have much varied negative predictive value of 62.5% with MTB64 gene, 78.74% with 19 kDa antigen gene and TRC4 element and 80.64% with 32 kDa protein gene. The positive predictive value of multi-gene PCR was 100% (tabulated in **Table 6**). Of four different methods used, histopathology and culture on L-J egg media showed false positive in 1 (0.33%) tissue sediment. On other side, 24 (7.95%) specimens showed false negative with 32 kDa protein gene and 27 (8.94%) tissue specimens were false negative with 19 kDa antigen gene and TRC4 element and 60 (19.86%) with MPB64 antigen gene by multi-gene PCR method. 98 (32.45%) tissue specimens were false negative by H & E staining, 158 (52.31%) tissue sediments were false negative by Z-N staining, 117 (38.74%) specimens were false negative with cultures on L-J egg medium and 121 (40.06%) cultures were false negative by Z-N staining. All TB suspects who were negative by multi gene PCR were infertile. All false negative samples evidenced by conventional and molecular methods were likely to represent negative for tuberculosis as multi-gene PCR was repeatedly proven negative, though, the patients were categorised as suspected cases of FGTB. In the non TB control group, all the tests were negative for TB. These results showed that the molecular method is more accurate than the conventional methods [18], [19], [45]. The results mentioned here demonstrate that *M. tuberculosis* was present in sufficient density among samples and can be detected surety by multi-gene PCR.

**Table 5:** Statistical significance tests between conventional and molecular methods (n = 302).

Methods versus Characteristics	Cases of FGTB (n = 202	Controls without TB (n = 100)	Pearson Chi-Square Value	Exact Significance (2-sided) Values	
				Fisher's Exact Test	McNemar's Test^a^
H & E Staining	104	1	75.167^b^	0.000	0.779
AFB+Ve on Z-N Staining of tissue sediments	44	0	25.497^c^	0.000	0.000
L-J Egg medium	85	1	55.418^d^	0.000	0.338
AFB+Ve on Z-N Staining of culture	81	0	55.725^e^	0.000	0.208
19 kDa (131 bp)	175	0	206.011^f^	0.000	0.000
TRC4 (173 bp)	175	0	206.011^g^	0.000	0.000
MPB64 (240 bp)	142	0	132.686^h^	0.000	0.008
32 kDa (506 bp)	178	0	214.612^i^	0.000	0.000

**Note**: “a” denotes binomial distribution used. Statistical analysis showed 0 cells (0.0%) have expected count less than 5 for all methods. Variation is observed for different methods in terms of the minimum expected counts (MEC), i.e. MEC for “b” is 34.77; “c” is 14.57; “d” is 28.48; “e” is 27.15; “f” is 42.05; “g” is 42.05; “h” is 47.02; and “i” is 41.06. Degree of freedom (df) is one (1) for all calculations. Data is considered statistically significant if p value is less than 0.05.

**Abbreviations**: n: number of patients; %: percentage; H & E staining: Hematoxylin and Eosin; L-J egg medium: Lowenstein Jensen; AFB+Ve: Acid fast bacilli positive; Z-N staining: Ziehl-Neelsen's; PCR: Polymerase Chain Reaction; 19 kDa (131 bp):19 kDa antigen gene; TRC4 (173 bp): TRC4 element; MPB64 (240 bp): MPB64 antigen gene; 32 kDa (506 bp): 32 kDa protein (MTP-59 α-antigen) gene.

**Table 6:** Comparative analysis of methods for detection of FGTB cases-control groups (n = 302).

Methods versus Characteristics	Positive Predictive Value (%)	Negative Predictive Value (%)	Sensitivity (%)	Specificity (%)
19 kDa antigen (131 bp)	100	78.74	86.63	100
TRC4 element (173 bp)	100	78.74	86.63	100
MPB64 antigen (240 bp)	100	62.5	70.29	100
32 kDa Protein (506 bp)	100	80.64	88.12	100
H & E staining	99.04	50.25	51.48	99
Z-N staining of tissue sediment	100	38.75	21.78	100
Culture on L-J Egg medium	98.83	45.83	42.08	99
Z-N staining of culture	100	45.24	40.1	100

**Abbreviations**: Multi-gene PCR: Multi-gene/multi-primer Polymerase Chain Reaction; H & E staining: Hematoxylin and Eosin; L-J egg medium: Lowenstein-Jensen; AFB+Ve: Acid fast bacilli positive; Z-N staining: Ziehl-Neelsen's; PCR: Polymerase Chain Reaction; 19 kDa (131 bp): 19 kDa antigen gene; TRC4 (173 bp): TRC4 element; MPB64 (240 bp): MPB64 antigen gene; 32 kDa (506 bp): 32 kDa protein (MTP-59 α-antigen) gene. FGTB: female genital tuberculosis. The following terms were described based on demographic details, clinical symptoms, and hysteroscopic/laparoscopic findings of infertile women highly suspected with female genital tuberculosis (FGTB) and control women (without tuberculosis): **True Positive**: Infertile women with asymptomatic clinical presentations; radiologically may or may not be indicating tuberculosis (TB); positive diagnosis of TB on laparoscopic examinations; Indication of proliferative solid epitheliod granulomas, dense polymorphornuclear cells etc on histopathology; demonstration of acid fast bacilli in culture, Z-N staining of menstrual blood fluids, pelvic aspirated fluids and endo-ovarian tissue biopsies; detection of mycobacetrial DNA on multi gene PCR; and positive response to assisted reproductive technology (ART) after anti TB therapy among suspected FGTB cases. **True Negatives**: Fertile and healthy women; radiologically negative for TB; normal diagnosis (without TB) or absence of abnormal clinical findings on laparoscopic examinations; normal or negative finding on histopathology; absence of tubercle bacilli in culture and AFB negative on Z-N staining of menstrual blood or pelvic aspirated fluids or endo-ovarian tissue biopsies; absence of mycobacetrial DNA on multi gene PCR; Anti TB therapy is not required. **False Positive**: Detection of TB among control women (group without TB) by conventional and molecular methods; detection of TB among fertile and healthy women/negative findings of TB on laparoscopic examination. **False Negative**: Detection of negative TB by conventional and molecular methods among infertile asymptomatic women highly suspected of FGTB and among women diagnosed with positive TB on laparoscopic examinations.

## Discussion

Female genital tuberculosis is an indefinable diagnosis and a high index of suspicion is very essential in the routine diagnostic process [59]. It is an important cause of infertility and may simulate advanced ovarian malignancy [60]. The risk of developing the disease can be observed among immuno-compromised individuals, including patients with severe psychiatric dysfunction, multiple sclerosis and other autoimmune disorders, women with diabetes, malnutrition and other medical disorders like hypertension [61], [62]. The signs, symptoms and altered immune responses of FGTB patients mimic those of immuno-suppressed individuals, so diagnostic awareness may prevent unnecessary treatment and morbidity [63]. That too, they pose the problem of diverting diagnosis and making treatment more complicated even though, they are not interfering with the PCR methods used for the detection of bacilli. In this context, our study is only on subclinical infertile patients, who are fully asymptomatic and with frank tuberculosis; which can be supported by assisted reproductive technology (ART) subsequent to anti-tubercular chemotherapy.

However, as there is no gold standard method in diagnosing FGTB and to compare multi-gene PCR, one should use a combination of microbiological, histological and radiological techniques [64], [65]. Laparoscopy is invasive, often nonspecific and expensive procedure by which absolute diagnosis of FGTB cannot be made, although laparoscopy can pick up the signs of disease and provides an opportunity to take the samples for laboratory investigations from various suspicious sites [21], [22], [64]–[67]. Clinical presentations such as peritubal adhesions, tubercles on the tubes and small tubo-ovarian masses were commonly seen in chronic cases. Distinctive diagnostic challenges such as subtle clinical manifestations were also over looked by laparoscopy during early stages of infections [9]. Several PCR techniques have been developed for the direct detection of *M. tuberculosis* [18], [19], [47], [68] and for the detection of *Mycobacterium* spp [47], [50], [69]. However, PCR using single target gene alone is not sufficient in the detection of all strains of *M. tuberculosis*, therefore use of multiple target genes is more appropriate [43], [45]. It has great potential in the laboratory diagnosis of FGTB, particularly in latent and paucibacillary conditions as well as in active tuberculosis. Improvement in the sensitivity of PCR was reported by using different set of targets in the detection of extrapulmonary tuberculosis [43], [44], [49]. However, one should use varied combination of parameters (or genes) to overcome the limitations such as the false positivity by way of contaminations, false negativity, dead bacilli and asymptomatic TB at different sites. Thus, the increase in PCR sensitivity and decrease in false negative results were achieved using dual targets for the detection of *M. tuberculosis* [58]. Even technical considerations, such as the use of suitable controls, standard strains, suitable conditions and the retesting of doubtful positive samples considerably influence the sensitivity and specificity.

The high endemicity of TB in India raises the possibility of FGTB patients harbouring a latent infection. A broad range of mycobacterial species are involved in causing such infections, but the type of complexes, species or type of strains that causes the disease are not clear. Identification of nontuberculous *mycobacteria* and treatments based on inconclusive findings involving different strains may not be adequate to control the disease. Therefore, this prospective large case-control cohort study was commenced for the detection of FGTB in Indian population for the first time. In the present study, we have demonstrated the use of a multi-gene PCR system based on the simultaneous amplification of the species-specific 19 kDa antigen gene, MTB complex specific TRC4 element and MPB64 antigen gene and genus specific 32 kDa protein gene in a single tube- single step reaction, by which MTB can be identified and distinguished from other nontuberculous mycobacteria. 19 kDa antigen (lpqH/Rv3763) gene [29], [70], TRC4 element (located in ORF Rv0697) [29], [71] and MPB64 protein gene [72]–[74] shared the property of being markedly species specific, widespread in mycobacterial genome [29] and routinely used in the diagnosis of FGTB. However, multi-gene PCR method may be limited in the diagnosis, since TRC4 element is there in all South Indian specific MTB complexes and it may not be suitable for other population. According to Sujatha *et al*, a huge number of clinical isolates of *M. tuberculosis* from South India had either a single copy (40%) or no copy (4%) of IS6110 [43], [44]. In such cases, the use of the multi-gene PCR system would not have the pitfalls caused by the absence of TRC4 element and IS6110 element from the particular region and mycobacteria, since DNA fragments corresponding to the amplification of 19 kDa antigen gene, MPB64 antigen gene and 32 kDa protein gene would still be present. The advantage of discriminating *M. tuberculosis* from nontuberculous mycobacteria in a single tube-single step reaction would lie in the possibility of using 19 kDa antigen gene and the TRC4 element, to identify the particular type of mycobacteria under the surveillance. 32 kDa protein gene showed higher sensitivity and specificity than any other gene due to the presence of infectious organisms representing *Mycobacterium* genus. Even 19 kDa antigen gene and TRC4 element showed better sensitivity (86.63%) and specificity (100%) indicating the infection due to MTB complex [47], [58]. Thus, these genes could be emerging future diagnostic biomarkers in the detection of FGTB [42], [43]. These results are similar to those at *Mycobacterium* genus and *M. tuberculosis* complex levels obtained in fine needle aspirates in 35 (87.5%) of the 40 patients with clinical and cytological diagnosis of tuberculous lymphadenitis [18], [47] and others by using single step PCR [45], [47].

MPB64 antigen gene could be appropriate or useful in the detection of *M. tuberculosis* complex but our results revealed that the role of MPB64 antigen gene is limited in the detection of *M. Bovis* among patients with FGTB. This may suggest mutations within the MPB64 gene, leading to the production of an incomplete protein as a result of deletion in the C-terminal region of the protein [75]. The results of this study inveterate that TRC4 element might be universally detected, especially among so called South Indian strain of *M. tuberculosis* [69]. Hence, the combination of 19 kDa antigen gene with TRC4 element could be a better choice in the detection of FGTB using endometrial tissue biopsy, ovarian tissue biopsy and pelvic aspirated fluids and the positive multi-gene PCR results can be given due importance. Increasing the awareness and importance of incorporating multiple genes, targeting different characteristics of infectious agents in a single tube-single step reaction using multi-gene PCR method is needed. Information of this investigation will be made available online, particularly in cases, where the data is not published. Culturing of decontaminated and concentrated (D & C) tissue sediments on L-J egg media and subsequent staining with Z-N stain have been showed significant increase in AFB positive cases than direct staining of D & C tissue specimens. Thus, culturing of tissue specimen was recommended prior to report the samples as AFB negative. Our results clearly demonstrated that multi-gene PCR showed a significant advantage over the conventional techniques, in that the minimal detection limits of bacilli without the use of radioisotopes, without the use of costly and complex equipments and more than that three target genes can be studied at a time [47]. Advanced nucleic acid-based methods such as multi gene PCR can be utilized not only for bacteriological presence but also in the clinical findings of host in response to infectious agents. This method also reveals that mycobacterial DNA is consistently observed more in ETBs and OTBs. Signifying that, the presence of localized and latent tuberculosis infections such as FGTB can be detected by taking the site specific sampling, irrespective of sample types. It seems improbable that reporting of FGTB would result in a spurious over-representation of women with infertility. Thus, supporting the use of endometrial tissue biopsy, ovarian tissue biopsy, endometrial aspirations, pelvic aspirated fluids and fluid samples from the pouch of Douglas (POD), therefore endorses the study by Bhanu *et al* on the importance of multiple sampling in aiding the diagnosis of FGTB [18], [19], [47]. In conclusion, multi-gene PCR was found to be a powerful technique for the diagnosis and differentiation of mycobacterial infections. Since 32 kDa protein is encoded by *Mycobacterium* genus specific gene, we suggest amplification of the 19 kDa antigen gene in combination with TRC4 element as a successful multi-gene PCR method for the diagnosis of FGTB among infertile patients using both cultured and uncultured endometrial tissue biopsies, ovarian tissue biopsies and pelvic aspirated fluids.
